# Treatment of Pleurisy

**Published:** 1845-02

**Authors:** 


Professor Mütter, in his introductory lecture, gives the following treatment in pleurisy, pursued by Trousseau, of Paris:

"The operation is nothing more than the evacuation of the fluid in cases of acute pleurisy, by an opening made into the thorax by the following process: 'A small incision is made in the skin, between the seventh and eighth ribs, a little to the outside of the heart. The *skin* is next raised until the incision corresponds to the intercostal space immediately above, and then an ordinary abdominal trocar is introduced to the depth of about two inches. On the spear being withdrawn the fluid rushes out, and in order to prevent the introduction of air into the chest, the *pavilion* of the canula is wrapt with a strip of bladder or gold-beater's skin, which is raised by the fluid as it passes out, but which falls on the orifice during deep inspiration, and effectually closes it. During the discharge of the fluid, an assistant compresses the abdomen so as to push up the diaphragm and thoracic parietes—and after its escape, the canula is rapidly withdrawn, the incision pushed down to its original position, and closed with a small piece of adhesive plaster.'"

